# Can T2 weighted imaging in cardiac MRI chest pain protocols improve diagnostic capability?

**DOI:** 10.1186/1532-429X-16-S1-P262

**Published:** 2014-01-16

**Authors:** Julian Kley, Brandon M Mikolich, John Lisko, Nicholas C Boniface, J Ronald Mikolich

**Affiliations:** 1 NEOMED, Rootstown, Ohio, USA; 2 Sharon Regional Health System, Sharon, Pennsylvania, USA

## Background

Evaluation of chest pain(CP) patients with cardiac MRI (CMR) provides a comprehensive exam including myocardial structure, function, perfusion and viability in 30-35 min without radiation or iodinated contrast. Patients who present with CP, but no ECG changes and normal biomarker levels are candidates for CMR evaluation. This study was designed to assess the diagnostic value of T2 weighted imaging, incorporated into a standard CMR chest pain protocol.

## Methods

All patients who underwent a CMR exam for CP or myocardial ischemia were extracted from an institutional CMR database, and constituted the study population. A policy to include T2 weighted imaging pulse sequences into the CP protocol was implemented in January 2012. Specifically, the incidence of pericardial disease was assessed before and after the protocol change. Presence of pericardial edema, pericardial effusion, pericardial thickening and pericardial late gadolinium enhancement(LGE) were tabulated. Pericardial edema was classified as patchy or diffuse, as was pericardial LGE. Pericardial effusion was classified as small, medium or large.

## Results

The diagnosis of acute pericarditis increased from 0.5% (5 of 995 patients) before T2 weighted edema imaging to 8.4% (49 of 583 patients) after T2 imaging was implemented by protocol. 2.4%(1 of 49) of acute pericarditis cases revealed diffuse pericardial edema, while 98.0%(48 of 49) showed patchy edema. 74.1% of acute pericarditis cases were associated with evidence of pericardial effusion, while 25.9% had no effusion. Only 7.3%(73 of 995) patients demonstrated pericardial LGE prior to the policy change, while 23.3%(136 of 583) had pericardial LGE after policy implementation.

## Conclusions

About 8%of patients who present for CMR evaluation of CP have evidence of acute pericarditis, which may go undetected if T2 weighted imaging is not incorporated into the CMR protocol for CP. This represents a 10-fold increase in detection of pericardial disease in patients with CP. Since pericardial edema is nearly impossible to detect with other imaging modalities, patients with CP due to pericarditis may go undetected with nuclear or echo imaging techniques, especially since 25% of patients with acute pericarditis have no effusion. CMR offers a novel approach to further diagnose patients with CP efficiently, without radiation exposure or obligate need for gadolinium.

## Funding

None.

